# Not Dreamt of in Horatio’s Philosophy

**Published:** 1894-04

**Authors:** 


### NOT DREAMT OF IN HORATIO'S PHILOSOPHY.

Here is not only only something new but something very interesting. It is a discovery of importance. It is not a theory exactly, but as yet, it can hardly be said to be a fact. It is a plausible explanation of the reason why a person,—why all animals in fact,—go to sleep, and why they wake up. It is a very handy explanation, inasmuch as it is made to do service in another important cause, and explain why it is that a person, however much accustomed to take chloroform, will, may be, die, during its administration. Occasionally such cases occur, and the profession, heretofore, have been unable to explain the cause of death. A case occurred in Austin recently where a man who appeared, from examination, to be a good subject for chloroform, died suddenly, during the administration of a small quantity of chloroform, much to the surprise ofthe doctors.

Dr. T. Lauder Brunton, who, it will be remembered, was a member of the celebrated Hyderabad Chloroform Commission, has written an article on the "Progress of Pharmacy," and published it in the *Humanitarian*, Mrs. Victoria Woodhull's English Journal of Sociology—(what a remarkable selection Mr. Brunton made for the publication of an article on *Pharmacy!*)—in which, after telling us how that, after all, the toad's poison is not poisonous,—but will cure dropsy by its stimulating action on the heart, branches off and says:

"It was just about the beginning of the present century that the first alkaloid, morphia, was discovered: * * * Ever since this discovery the number of alkaloids isolated from plants has been steadily increasing; but of late years chemists have not been content with simply obtaining new alkaloids from plants. They have set to work to make them artificially. Perhaps they have not been quite so successful as had been anticipated, but in the effort to make them, numerous bodies have been manufactured which are becoming of very great use in medicine. So numerous, indeed, are they and so fast are they increasing that it is becoming very hard work to keep one's knowledge of them abreast with the times, and a list of new remedies not very many months old is already antiquated. But, great as this department of chemistry is, there is another equally important, which appears to be just coming to the front. I mean the formation of alkaloids in the bodies of animals and of men. We know already that plants frequently contain more than one alkaloid, and that these (jaborandi for example) sometimes have an antagonistic physiological action. Others, again, such as nux vomica, contain two alkalies which have a similar action and will assist each other. Now alkaloids appear to be formed in the animal body, and these have not always the same physiological action. It would appear, for example, that during the day substances having a morphine-like action are formed more quickly than they are excreted, so that towards night the accumulation of these narcotic bodies tends to produce slumber, and thus the individual goes to sleep for the night. But during sleep a different set of substances is produced which have a stimulant action, and as these go on accumulating whilst the narcotic substances are being excreted the sleep becomes lighter and lighter, until at last the stimulant action gets the upper hand and the person awakes. Now it is evident that just as the alkaloids derived from plants may antagonize each other, so the alkaloids formed in the body may more or less completely antagonize the action of alkaloids given as medicines; and indeed experience by the bedside has long ago shown that the best time to give a narcotic is in the evening, when sleep would naturally occur of itself. We have been accustomed hitherto to look far too exclusively to the action of a drug, forgetting altogether that the result which it produces in a living body is the reaction between the drug itself and the organism. We have to deal, not with one factor but with two, and just as the result may be varied by altering the remedy administered, so it may also be changed by altering the body of the recipient. A great deal has been written lately in the medical journals about deaths from anæsthetics, and especially from chloroform, and the utmost care is now used to obtain anæsthetics free from impurity, because impurities have been looked upon, and probably rightly, as being responsible for some deaths. But it is quite possible that the impurity, if we may so term it, is not always to be found in the chloroform administered, but actually exists in the body in the form of alkaloidal substances, which in combination with chloroform tend to produce death.

"Lately Professor Poehl, of St. Petersburg, was visiting this country, and he informed me that in Russia they are now beginning to pay much attention to this subject, and they are now able, by analysing the urine beforehand, to tell whether the administration of chloroform will be dangerous in any case or not. In a tolerably large proportion of the deaths recently recorded, the anæsthetic had previously been taken by the same persons with perfect safety. Why death should occur in such persons after a second or third administration has hitherto been a mystery, but it can now be readily understood on the supposition that from indigestion, imperfect action of the liver or some other cause, the alkaloids were more abundant at the time of the fatal administration than they were on the previous occasions. The idea which is now being worked out in Russia occurred to me several years ago, and therefore some of the experiments which were made by the Hyderabad Commission, of which I was a member, were performed with the object of ascertaining whether disease of the kidneys, induced by cantharides or the alteration in tissue change generally which is induced by phosphorus, would render the administration of chloroform more dangerous.

"Rich fields of new investigation, rich harvests of practical usefulness in relieving disease and prolonging life, are rapidly opening out, but how are these to be utilized? * * * Thanks to the liberality of the Pharmaceutical Society, guided by the wisdom of the President and Council, a research laboratory has now been established in England, which has already done most excellent work and given promise of still more in the future."

